# The Exchange of Informational Support in Online Health Communities at the Onset of the COVID-19 Pandemic: Content Analysis

**DOI:** 10.2196/27485

**Published:** 2021-07-22

**Authors:** Wesley Jong, Ou Stella Liang, Christopher C Yang

**Affiliations:** 1 College of Medicine Drexel University Philadelphia, PA United States; 2 College of Computing and Informatics Drexel University Philadelphia, PA United States

**Keywords:** COVID-19, informational support, online health, online health communities, health information, online platform, pandemic, social support

## Abstract

**Background:**

Online health communities (OHCs) provide social support for ongoing health-related problems. COVID-19, the disease caused by SARS-CoV-2, has been an acute and substantial stressor worldwide. The disease and its impact, especially in the beginning phases, left many people with questions about the nature, treatment, and prevention of COVID-19. Unlike typical chronic ailments discussed on OHCs, which are more established, COVID-19, at least at the onset of the pandemic, is distinct in that it lacks a consensus of clinical diagnosis and an existing community foundation.

**Objective:**

The study aims to investigate a newly formed OHC for COVID-19 to determine the topics and types of information exchange as well as the sources of information this community referenced during the early phases of the COVID-19 pandemic in the United States.

**Methods:**

A total of 357 posts from a COVID-19 OHC on the MedHelp platform were annotated according to an open-coding process. Participants’ engagement patterns, topics of posts, and sources of information were quantified.

**Results:**

Participants who offered informational support had a significantly higher percentage of responding more than once than those seeking information (*P*<.001). Among the information-seeking topics, symptoms and public health practice and psychological impacts were the most frequently discussed, with 26% (17/65) and 15% (10/65) of posts, respectively. Most informational support was expressed through feedback/opinion (181/220, 82.3%). Additionally, the most frequently referenced source of information was news outlets/websites, at 55% (11/20). Governmental websites were referenced less frequently.

**Conclusions:**

The trends of this community could be useful in prioritizing public health responses to address the most common questions asked by the public during crisis communication and in identifying which venue of communication is most effective in reaching a public audience during such times.

## Introduction

### Background

Since the start of the COVID-19 pandemic, the disease has been a topic of unceasing concern worldwide. The onset of SARS-CoV-2 created many uncertainties, particularly pertaining to the epidemiology of the virus and its impact on people. Especially in the beginning phases of the SARS-CoV-2 epidemic, the symptoms and severity of the disease varied from person to person, and the transmission of the virus was not well understood [1]. The impact of COVID-19 has been shown to cause psychological distress by vicarious trauma not only among health care workers but also in members of the general public [2]. As the novel circumstances created by COVID-19 evolve, these unknown factors continue to be a point of discussion and revelation in efforts to mitigate health concerns and apprehension among the public [3]. Coping with the effects of COVID-19 has become a new challenge globally, and one coping method among many is seeking social support [4]. With the ongoing pandemic, efforts to disseminate and provide support have become increasingly important to offer solace and guidance.

Particularly, given the current climate, transitioning many aspects of pre–COVID-19 life to a web-based format has become a movement in itself. With the shift to virtual classrooms, conferences, and clinics (telemedicine), the emphasis on the internet is as dominant as ever. Online support communities offer accessibility to provide comfort to those who are seeking it. Historically, online health communities (OHCs) or forums have been used as a platform for a variety of conditions, particularly chronic diseases. OHCs provide empathic peer-to-peer support by giving participants a safe space to offer shared connections and emotional understanding [5]. In addition to the emotional aspect of social support, these communities provide informational support to those who are seeking advice [3]. Analysis of the interaction within these communities has provided insight about information exchange and behaviors for many established diseases [6,7].

However, given the novelty and impact of COVID-19, the response of COVID-19 support communities may not be similar to those of established diseases. The departure from the norm of chronic diseases presents a unique opportunity to observe the needs of this community (eg, where participants are getting their information and how outlets of information may be directed in these scenarios in the future). The presence of these communities dedicated to COVID-19 appears to have a wide spectrum of focus and social support. In social media platforms such as Reddit, many public health issues have emerged as popular topics for discussion [8]. However, the public nature of these popular platforms makes them susceptible to an *infodemic*, or the spread of misinformation across media [9]. In contrast, dedicated OHCs, such as MedHelp, offer expert-moderated content to improve the accuracy of information. The participants are typically patients, caretakers, and health care professionals, who may form another layer of resistance to misinformation. Research has been active on popular social media platforms (eg, Twitter and Weibo) and COVID-19 [10-15]; however, there appears to be a gap in knowledge about how established, health-tailored communities have been responding to COVID-19. For these reasons, we will focus on a new COVID-19 community on MedHelp.org [16] for this study.

Social support can be organized into four broad types of supportive behaviors: emotional, instrumental, informational, and appraisal support. These behaviors are not mutually exclusive and may coexist in a single social exchange [17-19]. Bates [20] argues that information-seeking behavior is not only social and cultural but is also embedded in the biological and physical anthropological layers of human existence. In the context of COVID-19, investigating people’s health concerns and informational needs is particularly important to determine actionable steps to provide reassurance and safety at the emergence of a previously unknown disease. By examining the originating posts in this OHC, our goal is to identify the topics of information that the participants are seeking. Additionally, studying the types of informational support in the form of responding posts would give a sense of how members of this community are interpreting the pandemic as a whole and how they are engaging and managing the information around them. In the participants’ responses, the sources of information would help provide a better understanding of where people are receiving most of their information and what resources might be lacking in delivering patient education materials. Especially given the accompanying infodemic, investigating where most sources are referred to would help formulate possible future directives for information dissemination. To summarize, we aim to address three research questions (RQs) through our investigation:

1. What patterns of engagement did participants have in the newly formed OHC?
2. What were the topics of information-seeking posts and types of informational support?
3. What sources of information were referenced most frequently?

### Prior Work

#### Social Support in OHCs

There is a robust body of literature investigating social support in online health forums or communities. Many are related to chronic health conditions such as cancer, diabetes, or substance use disorder [5,7,21-23]. These established diseases are typically in chronic care or require a degree of maintenance. Acute episodes are possible, but the overall projection is long-term; thus, attention should be paid to factors beyond physical medical treatment, such as the psychological implications, which are garnered through social support.

In terms of the nature of social support in online communities for health causes, Coulson [24] examined five thematic social support categories: emotion, esteem, information, network, and tangible assistance. Among these categories, informational support was used the most for areas of symptom interpretation, illness management, and interaction with health care providers [24]. Online support for alcoholism in an OHC showed subcategories of informational support that included advice, referral, fact, personal experience, and opinions; facts were the most frequently exchanged [6,25]. Additional studies show that informational and emotional support is the most frequently offered form of social support and is key to the functioning of online groups [26,27]. In investigating the patterns of social support exchange between OHC participants, Zhang and Yang identified four behaviors, including active giving, active receiving, passive giving, and passive receiving [7]. Empathy analysis of OHCs demonstrated that empathy develops through shared experiences [22], and empathy was perceived through effectiveness of information seeking rather than general social support [28]. Broader functions served by general-purpose online social platforms include raising awareness, fundraising, and commercial promotional content [5,21].

#### Information Studies Related to COVID-19

As COVID-19 quickly spread in 2020, an increasing amount of research work was performed to understand how the public was responding to the pandemic by analyzing social media data. Applications of qualitative and quantitative methods to topic identification and modeling were the most common studies, and general-purpose microblogging sites such as Twitter and Weibo served as much of the research corpora. One study identified the top concerns among Twitter users to be the origin of the virus; its sources; its impact on people and society; and ways of mitigating the risk of infection [29]. Xue et al [14] used latent Dirichlet allocation to identify popular unigrams and bigrams representative of salient topics and sentiments in the collected COVID-19 tweets, and they found that confirmed cases and death rates, preventive measures, health authorities and government policies, COVID-19 stigma, and negative psychological reactions (eg, fear) were the dominating topics on Twitter [14]. Chang et al [12] developed online non-negative matrix factorization algorithms to detect the evolving COVID-19 topics over time on Twitter; government policy, economic crisis, COVID-19-related updates and events, prevention, vaccines and treatments, and COVID-19 testing were some of the most important evolving topics identified. Zhao et al [15] explored the types of information most frequently searched by Chinese netizens during the pandemic on Weibo: accessing medical treatment, confirmatory testing, managing self-quarantine, and offline-to-online support.

The public sentiment during the pandemic is another area of focus. Boon-Itt and Skunkan [11] found that Twitter users had a negative outlook towards COVID-19, and fear was the most frequent negative sentiment. Lwin et al [13] found that the emotions of the public shifted from fear to anger over the course of the COVID-19 pandemic on Twitter, and sadness and joy began to surface as people lost loved ones or expressed gratitude and hope for recovery.

In addition to topic and sentiment analysis, social media data were also analyzed for syndromic surveillance, fulfilling the notion of infodemiology [30]. Alanazi et al [10] collected tweets about COVID-19 and found that the 3 most commonly mentioned symptoms were fever, headache, and anosmia. Researchers in China analyzed the symptom descriptions and clinical test results posted voluntarily by Weibo users [31].

Finally, a limited number of studies sought to analyze the characteristics of the information posted on the web, such as its validity and patterns of spreading. Jo et al [32] identified the topics and appropriateness of questions related to COVID-19 at the early stage of the outbreak posted on a popular Q&A web forum (Naver Jisik-In) in South Korea; they concluded that the answers to suspected physical symptoms were relatively accurate, but a high proportion of answers related to self-protection methods contained misinformation or advertisement content. Park et al [33] studied how COVID-19–related news articles circulated on Twitter in Korea; they found that the choice of words for referencing the disease affects the speed of information spread, and medical-themed articles are more popular than nonmedical reporting of the disease.

To summarize, research on COVID-19–related web-based discussions published to date has primarily focused on identifying the topics and public sentiment reflected by the content. The identified topics, while informative, are diverse and lack a common framework to generalize for future public health emergency planning. Furthermore, these studies used general-purpose social media platforms, whose content may be generated by news publishing organizations, commercial accounts, or special interest groups that are not representative of the average health consumers. Meanwhile, studies on social support seeking as a means of disease management have been abundantly studied for many existing health conditions, our understanding of how the public seeks support in the face of an emerging pandemic is limited. In this study, we focus on the characteristics of the informational support exchange related to COVID-19 among OHC participants. In particular, we investigate the patterns of participation, topics of information seeking, types of informational support, and sources of information referenced.

## Methods

### Data Source

We collected data from MedHelp [16], an online health and wellness forum with more than 150 support communities dedicated to individual health topics, regarding COVID-19 discussions between March 12 and June 25, 2020. COVID-19 began to significantly impact life in the United States in March 2020, and this month is also when the MedHelp COVID-19 community began its activity. The unit of analysis is a post. During this time, there were a total of 83 originating posts and 274 responding posts. The originating posts were questions raised by participants who were seeking information. The responding posts were answers offered by other participants. In addition to the responding posts, participants were able to provide comments on the responses, which were excluded because they may not have a direct relation to their corresponding post. All data collected are publicly and freely accessible on the internet. 

### Data Analysis

Qualitative content analysis was performed on posts for information seeking and informational support. The posts and responses were exported from the platform into an Excel file (Microsoft Corporation). The variables in this document included a numerical ID of the post, the post topic, the post content, the post creator, the post date, a numerical ID for the response, the response topic, the response content, and the response creator. These variables were then reviewed and coded by one researcher, who is a medical student. Annotations included categorizing the topics of information-seeking posts, the types of informational support responses, and the sources of information for referral posts. The annotated results were randomly sampled and reviewed by another researcher with experience in qualitative data analysis. The researchers discussed the ontology and clarified concepts that might fall under multiple categories. For example, posts inquiring about mask-wearing can be categorized under transmission, protection, and public health practices and psychological impacts, but we focused on their different emphases: transmission is about people wanting to understand the mechanism underlying how a particular protection measure might work; protection is about seeking information on a specific protective measure; and public health practices and psychological impacts is about building consensus on protective practices for group well-being. The coding definitions and examples are provided below. The frequency of each of the topics for information seeking and the types of informational support were then quantified.

#### Topics of Information Seeking

To understand participants’ inquiries about different aspects of COVID-19, the topics of information seeking were coded for the 83 originating posts. Common topics were identified based on the subject matter and context of the post. These included health risk, symptoms, transmission, prevention, prognosis, protocols, disease management, and public health and psychological impacts (Table 1).

**Table 1:** Topics of information seeking, their definitions, and examples.

Name of topic	Definition of topic	Example posts
Health risk	Having a notable past medical history that includes pre-existing conditions, such as diabetes, lupus, and cancer, or past traumatic events, such as hospitalizations and treatments	“I had a septic blood disease 5 years ago which caused spots on my lung and my brain. I was hospitalized for 32 days over the course of three months...Does this make my immune system more susceptible to catching the coronavirus at this time?...” “So in layman's terms, who is high risk? Are people of a certain age automatically high risk, even if we're healthy?...” “I’m in my 70s, but healthy. If I don’t have diabetes, heart disease or lung issues, do I have to stay inside?”
Symptoms	Specific characteristics that are relevant to the presentation of COVID-19, such as cough and loss of smell. These also include differentiating factors from other similar disease presentations, such as influenza.	“I've had this left side throat pain for about 4-5 days now…I don't have any trouble breathing, stuffy/dripping nose, aches/pains, I'm not dry coughing and I'm not running a fever. Should I be worried about this?” “…I was diagnosed with sinusitis on Thursday... Monday morning I woke up with a low grade fever of 100.1 and a sore throat...I have no other symptoms...any advice?...” “Does the normal Flu [influenza] have SOB [shortness of breath]?” “…i've lost my smell and taste. Had a mild cough a few days before this. Is it covid19?...” “Covid Toes, what are they?”
Transmission	Means by which COVID-19 can be transferred, be passed on, or travel	“…I was washing produce that was brought from the grocery…and water splashed my face. My wife…mentions that's how this can spread…Is it possible that she may be right?” “My daughter ordered two tee shirts…I put them in the tub with detergent and scrubbed them…and some water splashed into my eye… She [received] the order in only 3 days. How long would it stay on it? And could the germs be that potent to get into my eye?” “…I saw a suspect 10 feet away while walking, he was asking the security guard for Covid 19 testing area. I did not go closer or touch. I was wearing face mask I came home and washed everything and sanitised my self by taking a bath. I'm i [*sic*] at any risk of catching the virus? Does it transmit through air?...”
Prevention	How to avert or avoid contracting the virus, or prophylactic measures taken to lessen the potential response to the body	“I'm interested in a discussion about how to keep my immune system top notch to help fight the corona covid 19 virus should I get it. Should we use more vitamin C? Drink fluids? Vitamin D? Suggestions?” “… I have been wearing masks when flu season starts, for many years.…So is K N95 the same as N95??” “Has there been any study or proof of breathing 1 to 2 deep breaths of diethyl ether fume to kill bacteria or viruses in nasal area or lungs.being as a preventive measure against getting the virus…”
Prognosis	The course of the disease, which includes the timeline, recovery, progression, outcomes, and lasting effects	“Can anyone who RECOVERED from Covid19 please post some info? The community would very much appreciate some actual details about the good, the bad, and the ugly. Is the situation so dire that no one can post details here?” “What is the expectation of longer term lung damage after COVID-19? My experience with the Hepatitis C has taught me a virus can leave its mark even after cured.” “How long do people actually have it? What is the typical recovery time?”
Protocols	The testing for the virus, which may include nasal swabbing, antibody testing, or questions about operations in handling specific scenarios	“How long after exposure would the virus be detected by a PCR [polymerase chain reaction] test?” “A nurse in a nursing home tested positive to covid 19. They had been in direct contact with residents on their unit. What should have been done was it was known that the nurse was positive?” “I…have a deviated septum and possibly some other structural differences in my nose…Could this affect whether the swab can be inserted far enough back to get enough of a sample for the test?”
Disease management	Handling of the disease, such as treatments, medications, therapies, and ventilator use	“Are there truly any medications or treatments for COVID/19?” “Would hyperbarics [*sic*] chambers oxygenize in a different way than ventilators, or is it the same thing?...” “…Why not avoid aggravating the lungs by “working with” the symptoms by filling the lungs with high-Oxygen liquid?...”
Public health practices and psychological impacts	Broad range of questions that stem from effects of COVID-19; public health concerns may vary from topics such as social distancing to quarantine/shelter-in-place. Psychological concerns involve discussion about anxiety and depression.	“There seems to be so much conflicting info on masks. Are you wearing one? Why or why not? What kind are you wearing, if you are?” “What are you personally going to do in order to protect you and your loved ones as so many locations begin coming back online?” “How are you all coping with the inevitable fear. Fear of our health, our finances, life changing forever. What are your coping strategies? Anything you are looking at in a new way now verses before?” “I'm really worried like I'm sure a lot of people are. Anxiety is running high. I'm also feeling really shut in and trapped due to social isolating and distancing. How are people handling this?”
Not applicable	Content that is not defined by the other topics of information seeking and is not directly relevant to health matters of COVID-19. These topics may include conversation starters and optimistic ideas. Topics that are not involved in direct information-seeking but are presented as a post are also included here. These may include references or resources that are not linked to specific information seeking.	“... What have you had positive come from this? Do you know a positive story?” “... Think any of the changes you are making will become new habits? Let me know what you think and which ones will be your new normal habit!” “Washington Examiner article excerpts below suggesting only 70% sensitivity. They don't mention specificity %. https://www.washingtonexaminer.com/news/health-experts-believe-1-in-3-infected-patients-getting-negative-coronavirus-test-results....” “https://www.upworthy.com/doctor-shares-potential-life-saving-coronavirus-breathing-technique”

#### Types of Informational Support

The study of the exchange of seeking and furnishing informational support is not complete without studying the participants’ responses to the originating posts. A total of 274 responses were reviewed and annotated following the types of informational support outlined by Chuang and Yang [6] and Cutrona and Suhr [34], including reference/referral, advice, feedback/opinion, facts, personal experience, and perceptual knowledge (Table 2).

Due to the circumstances of the COVID-19 pandemic, distinguishing the facts remains a challenge because of the many unknown factors, the unique presentations per person, and the fact that information about the disease is constantly changing. Therefore, the definition of *fact* based on previous literature is not applicable. Similarly, the definition of *perceptual knowledge* from previous literature cannot be applied in this context.

**Table 2:** Types of informational support, their definitions, and examples.

Name of support type	Definition of support type	Example post
Reference/referral	Responses that directly provide a source of information for the user to refer to. These responses also include sources or links embedded in a response.	“Results from new studies reported in livescience.com say…” “https://www.lupus.org/news/coronavirus-update-access-to-hydroxychloroquine-plaquenil-for-people-with-lupus”
Advice	Responses that offer suggestions to a specific problem or concern that a user may have	“we do want to let you know that if you can't breath [*sic*], you should seek immediate emergency care” “definitely talk to your oncologist when you face such an important question. Ask him or her if untreated cancer is more dangerous or if the chemo would be more dangerous for some reason.”
Feedback/opinion	Responses that reflect the responder’s judgment of a certain situation or idea. These include responses that are not directly referenced by a source but through general information heard about the disease summarized and given as information, interpretation of a reference or source, or interpretation of a situation.	“it's a blood clotting problem from what I've heard. This virus is weird. It affects different people very differently. It can adversely affect virtually every major organ in the body…” “I have read that this loss of smell and taste is definitely commonly reported as an early symptom. This virus has a lot involved with it. This is an easy one to spot.
Personal experience	Responses that are an anecdotal recounting of a user’s story to provide insight to a post. These may also include conditions relevant for support and reflections on their own experience handling the situation.	“I had the virus early April…” “I also get allergies when the weather changes…”
Fact	Responses that reassure the user about the facts of the disease	No instances found
Perceptual knowledge	Responses that provide sensory information to the user that helps reassess the situation	No instances found
Not applicable	Responses to originating posts that are in the “not applicable” category	“Fewer cars, clearer air. I also like getting some sleep.” “My hair has gotten longer in quarantine (the last appointment I canceled when we got word that we were about to go on lockdown was a haircut). When it's shorter I have to air style to look presentable...”

#### Sources of Information Referenced

We also documented the sources of information referenced in the responses. These sources were reviewed and defined through open coding and then categorized. Among the references, 6 categories were created to categorize the source of information. These categories are listed below.

1. News outlet/website: references to general news sources, health news, international news, etc
2. Government: references to governmental websites, such as the US Centers for Disease Control and Prevention
3. Medical journal: references to peer-reviewed journals, such as *The Lancet*
4. Health website: references to specific sites about diseases, such as lupus, cardiac disease, and COVID-19
5. World Health Organization: references to the World Health Organization (WHO)
6. Other: references to social media, specific product websites, or other MedHelp communities

## Results

### RQ1: What Patterns of Engagement Did Participants Have in This New Community?

In the newly formed OHC, participants established meaningful connections by creating and responding to posts about COVID-19 to seek information and offer support. A total of 78 participants contributed to information seeking and offering. Among them, 45% (36/78) only contributed to information seeking, 36% (27/78) contributed only to information offering, and 19% (15/78) contributed to both information seeking and offering.

**Figure 1:**
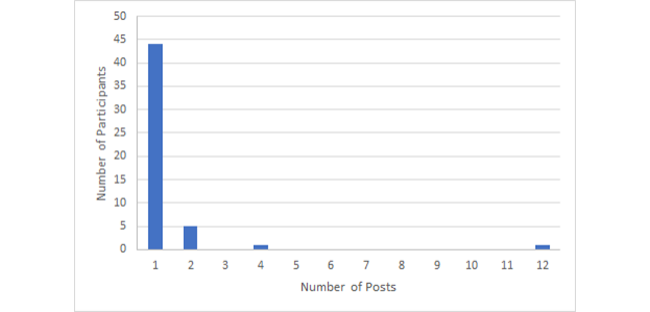
Histogram of participation frequency related to information seeking.

**Figure 2:**
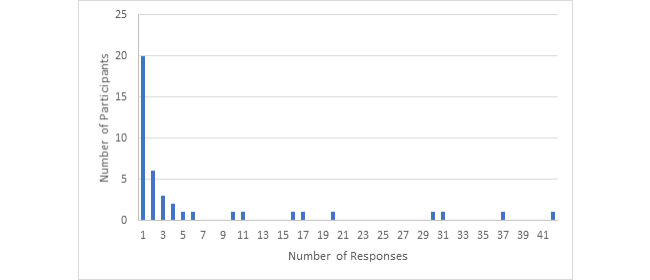
Histogram of participation frequency related to information offering.

Furthermore, among the 51 participants who sought information, the majority (86%, 44/51) posted only once, with 1 person making 12 posts (Figure 1). In comparison, a total of 42 participants contributed to information offering, among whom 20 (20/42, 48%) posted once, 14 (14/42, 33%) responded 2-10 times, 4 (4/42, 10%) responded 11-20 times, and 4 (4/42, 10%) responded more than 20 times, with the highest number of responses being 42 (Figure 2). Information seeking and offering by the participants demonstrated similar patterns in that most participants interacted with the community via only 1 thread of conversations, although those who offered information had a significantly higher percentage of responding more than once (*P*<.001).

### RQ2: What Were the Topics of Information-Seeking Posts and Types of Informational support?

The content of information seeking holds importance in evaluating the most pertinent information that needs to be addressed for the general public at the beginning of a pandemic. The responses to these posts are the informational support offered by the members of this community. The distribution of these responses gives insight to how members of this community are offering their support and which information-seeking type elicits the most conversation.

Out of the total 83 originating posts, 65 posts were relevant to participants seeking information. Among the information-seeking topics, symptoms were the most frequent (17/65, 26%), followed by public health practice and psychological impacts (10/65, 15%) and transmission (10/65, 15%) (Table 3).

**Table 3:** Distribution of the information-seeking types categorized into the number of information-seeking posts, the number of responses corresponding to the information-seeking category, and the response-to-post ratios.

Information-seeking topic	Posts (n=65), n (%)	Responses to posts (n=220), n (%)	Response-to-post ratio
Symptoms	17 (26.2)	32 (14.5)	1.9
Public health practice and psychological impacts	10 (15.4)	61 (27.7)	6.1
Transmission	10 (15.4)	28 (12.7)	2.8
Health risk	9 (13.8)	29 (13.2)	2.8
Disease management	7 (10.8)	23 (10.5)	3.3
Prognosis	5 (7.7)	22 (10)	4.4
Prevention	4 (6.2)	19 (8.6)	4.8
Protocol	3 (4.6)	6 (2.7)	2.0
Not applicable	18 (27.7)	54 (24.5)	N/A^a^

^a^N/A: not applicable.

Within the total 274 informational support responses, 220 responses correspond to informational support (Table 3). The most common informational support responses were related to public health practices and psychological impacts (61/220, 27.7%) followed by symptoms (32/220, 14.5%). There were similar distributions of transmission (28/220, 12.7%) and health risk (29/220, 13.2%) as the next most common categories. Disease management (23/220, 10.4%), prognosis (22/220, 10.0%), and prevention (19/220, 8.6%) were also generally evenly distributed among the total responses to information-seeking posts. The protocol topic had the lowest number of responses (6/220, 2.7%).

The number of responses to information seeking was compared with the number of originating posts in their corresponding categories to evaluate which information-seeking topics offered more discussion than others in terms of response-to-post ratio. Interestingly, the category of information seeking with the highest response-to-post ratio was public health practices and psychological impacts, with a ratio of 6.1 responses per post, while the lowest was symptoms, with a ratio of 1.9 responses per post. Public health practices and psychological impacts generated more discussion than symptoms; however, the latter had the highest number of information-seeking posts.

**Figure 3:**
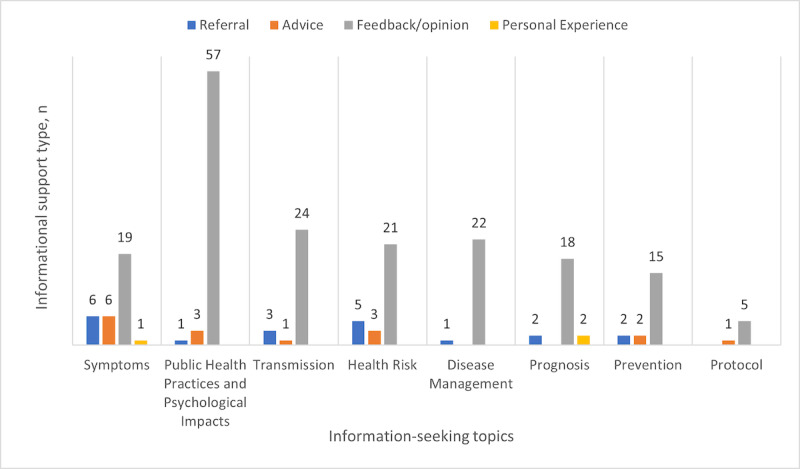
Distribution of information support for the subcategories of information seeking. The frequency of each is noted on top of the bar corresponding to its color.

Among the types of informational support, feedback/opinion was dominant, with 181 responses (181/220, 82.3%; Table 4). Within the feedback/opinion type, the majority (57/181, 31.5%) of responses addressed the topic of public health practices and psychological impacts (Figure 3). Within the topic of symptoms, feedback/opinion was still the most common type (19/32, 59%); moreover, compared to the other topics, symptoms received the most referrals (6/32, 19%) and advice (6/32, 19%). Prognosis and symptoms were the only topics that had personal experience responses (2/22, 9%, and 1/32, 3%, respectively). There were no responses for facts or perceptual knowledge.

**Table 4:** Frequency of the informational support responses.

Informational support type	Responses (n=220), n (%)
Feedback/opinion	181 (82.3)
Referral	20 (9)
Advice	16 (7.7)
Personal experience	3 (1.4)
Fact	0 (0)
Perceptual knowledge	0 (0)
Not applicable	54 (24.5)

### RQ3: What Sources of Information Were Referenced Most Frequently?

**Figure 4:**
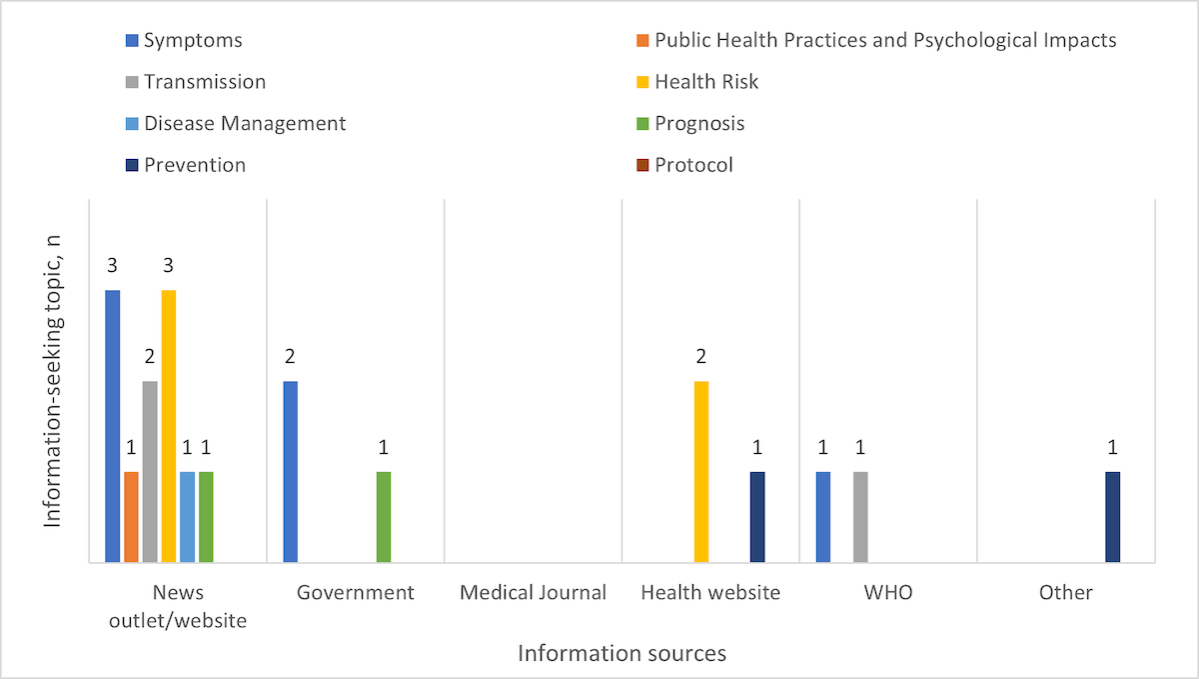
Distribution of sources of information by information-seeking topic. WHO: World Health Organization.

The different types of reference sources reflect how members of the MedHelp COVID-19 community were receiving their information and which venues they may have found to be relevant for informational support. A total of 20 responses corresponded to the reference/referral type of informational support, among which 11 references (55%) used news outlets/websites, 3 (15%) used governmental websites, 3 (15%) used health websites, 2 (10%) used information from the WHO, 1 (5%) used information from other sources, and none used information from medical journals (Figure 4).

Participants referenced news outlets/websites when responding to posts with the topics of symptoms, public health practices and psychological impacts, transmission, health risk, disease management, and prognosis. Governmental sites were referenced in the symptoms and prognosis subcategories. Health websites were referenced in the health risk and prevention subcategories. The WHO was referenced in the symptoms and transmission subcategories. Other sites were referenced for prevention only. There were no direct references to medical journals for information seeking posts, and no references were made to a protocol.

## Discussion

### Principal Results

In this study, we investigated the characteristics of a newly formed OHC dedicated to COVID-19, including participation patterns, topics of concern, and sources of information. A total of 78 participants generated 83 originating posts and 274 responses during a 3-month period at the onset of the COVID-19 pandemic in the United States. Within these posts, 65 posts were categorized as information-seeking and 220 responses were identified as offering informational support. Among the participants, 65% (51/78) sought information and 54% (42/78) provided informational support, with a large majority of information-seekers (44/51, 86%) and a slight minority of information providers (20/42, 48%) posting only once. The most common topic of information seeking was related to symptoms of COVID-19 (17/65, 26%), followed by public health practices and psychological impacts (10/65, 15%), and mechanisms of transmission (10/65, 15%). The topics that garnered the most responses were public health practices and psychological impacts (61/220, 27.7%), symptoms (32/220, 14.5%), and health risk (29/220, 13.2%). Among these popular topics, public health practice and psychological impacts saw the highest response-to-post ratio (6.1); symptoms had the lowest ratio, at 1.9 responses per originating post. Most informational support was in the form of feedback/opinion (181/220, 82.3%), which reflected the responder’s judgment of a certain situation, followed by information references as a distant second (20/220, 9.1%). The participants primarily relied on news outlets (11/20, 55%) as sources of information.

The participation trends reflect the power law distribution that is common in social networks, where the majority of participants may only contribute once or a few times and there are a few individuals with high numbers of posts (Figure 1 and Figure 2). A few considerations related to the reason for the greater activities of certain members are having a health care background, personally knowing someone infected with or at risk of COVID-19, familiarity with the platform, or other factors. In addition, some participants were members of multiple communities in MedHelp prior to COVID-19, who readily contributed social support in other communities.

The study shows that the general public may be most concerned with the symptoms and manifestation of a disease when confronted by a previously unknown disease at the beginning of an epidemic. Considering the timeframe of these posts, the highest frequency of information seeking in symptoms is understandable because during this time, there were many unknown factors regarding SARS-CoV-2 and COVID-19. Additionally, the devastation in previously affected countries may have led to the development of insecurities and fear in the public. Knowing that symptoms are the first signs of the manifestation of a disease, the frequency of inquiries about this category does seem to be the most reasonable finding given the public’s concern regarding their well-being and how certain symptoms present in association with the disease. Furthermore, symptoms also had the lowest response-to-post ratio, suggesting a paucity of relevant information among the public. Public health professionals may focus on educating the public about known symptoms to reduce the potential of misinformation.

Compared to symptoms, the topics of public health practice and psychological impacts were not only among the most requested topics but also received the highest response-to-post ratios. At the onset of the pandemic in the United States, various levels of health and safety measures were put in place by different states, possibly creating confusion and debate among the public about best practices (eg, whether mask-wearing is effective). Meanwhile, reports of the rising hospitalizations, the lack of protective gear and equipment, and a growing list of newly discovered complications may have taken a toll on the psychological well-being of the general public. The public health practice and psychological impacts of the pandemic were affecting the daily life and social activities of every person. Many participants were responding to this topic, and the majority of informational support was in the form of feedback and opinion. Out of 61 posts offering informational support to the public health practice and psychological impacts, there was only one reference to information from a news outlet or website.

The topic of *protocol* had the lowest number of posts, which may also be attributable to the timeline. With more information about the disease, there could be better means to expedite patient education information and to implement actions for testing and better management of containment. The responses being primarily driven by feedback/opinion reflects the lack of concrete information during this time as well. It is also possible that the general public views the protocols of testing and hospital operations as requiring the expertise of health care professionals and thus not an area of interest to discuss.

These trends could indicate that among the participants of this community, their concerns pertained not only to the pandemic itself but also to how the pandemic affected their daily lives. The low response-to-post ratio for symptoms could indicate that on one hand, the general public lacked the knowledge to offer support, and on the other hand, the posts for symptoms may have been phrased as recounts of individual circumstances to solicit reassurance, thereby leaving less space for a community discussion on what may be considered symptoms of COVID-19.

Feedback/opinion is the most frequent informational support type (181/220, 82.3%). It is provided as a respondent’s judgment without referencing any information source but only offering their opinions based on what they have heard or interpreted. This finding shows that there is a lack of authoritative information to support the community. Users are mainly relying on their own judgment to support others, and theirs interpretations of the information they acquire can be unreliable in some cases.

Referral was the second highest informational support type (20/220, 9.1%). Among all the information sources, news outlets and websites were the most frequently referenced information sources (11/20, 55%) by the participants. Governmental and WHO sources as references appear to be underused or insufficient when referencing circumstances surrounding this pandemic. The trends within this community may demonstrate where information dissemination is most effective. Possible reasons for the frequent use of new outlets and websites include memorable anecdotal accounts of the disease, more immediate coverage, and accessibility. With the changing guidelines in protocols, public health measures, and disease information, it is understandable that there is difficulty maintaining consistency in the shifting landscape. However, maintaining consistency with so many unknown factors and fluctuations is important for safety and reassurance. The news outlets and websites likely provided this community with reassurance and updates more reliably than the other types of sources.

### Limitations

Only one coder annotated the study. Although this is helpful in terms of consistency in annotation and interpretation, having more than one coder could have been beneficial in determining nuances in the contents of the posts and responses. The time frame of the study provides only a snapshot of the beginning of the disease progression and is not predictive of the course of how this platform will continue to respond as the disease progresses. The annotated posts do not reflect the views of people who visit the OHC without posting or responding.

### Conclusions

The MedHelp OHC for COVID-19 reflects real-time concerns during the pandemic. These concerns are important in understanding how OHCs facilitate the exchange of information at the onset of a pandemic. Among the information-seeking topics, interest in symptoms was highest, followed by the public health practices and psychological impacts. However, there was a higher number of responses per post for posts related to public health practices and psychological impacts compared to posts about symptoms. Feedback and opinion was the most frequent type of informational support, followed by referrals. The most referenced source of information referral was through news outlets/websites. Government websites and the WHO were less frequently used. The referral trends suggest that news outlets/websites are the most effective mode of communication that individuals can refer to. These findings may be useful in prioritizing public health responses to address the most common questions sought by the general public during crisis communication and in identifying which venue of communication is most effective in reaching the public audience during these times.

## Abbreviations

OHC: online health community
RQ: research question
WHO: World Health Organization
